# Spatial-Frequency-Scale Variational Autoencoder for Enhanced Flow Diagnostics of Schlieren Data

**DOI:** 10.3390/s25196233

**Published:** 2025-10-08

**Authors:** Ronghua Yang, Hao Wu, Rongfei Yang, Xingshuang Wu, Yifan Song, Meiying Lü, Mingrui Wang

**Affiliations:** 1 School of Civil Engineering, Chongqing University, Chongqing 400045, China; 202316021016@stu.cqu.edu.cn (H.W.); 202416131265t@stu.cqu.edu.cn (M.W.); 2 College of Energy and Power Engineering, Nanjing University of Aeronautics and Astronautics, Nanjing 200016, China; yrf@nuaa.edu.cn (R.Y.); wxsnuaa@nuaa.edu.cn (X.W.); 3 Shenzhen Institute of Advanced Technology (SIAT), Chinese Academy of Sciences, Shenzhen 518055, China; yf.song2@siat.ac.cn; 4 School of Mathematical Sciences, Chongqing Normal University, Chongqing 401331, China; lmy19831102@163.com

**Keywords:** schlieren imaging, optical sensing, variational autoencoders, unsupervised feature decomposition, flow diagnostics

## Abstract

Schlieren imaging is a powerful optical sensing technique that captures flow-induced refractive index gradients, offering valuable visual data for analyzing complex fluid dynamics. However, the large volume and structural complexity of the data generated by this sensor pose significant challenges for extracting key physical insights and performing efficient reconstruction and temporal prediction. In this study, we propose a Spatial-Frequency-Scale variational autoencoder (SFS-VAE), a deep learning framework designed for the unsupervised feature decomposition of Schlieren sensor data. To address the limitations of traditional β-variational autoencoder (β-VAE) in capturing complex flow regions, the Progressive Frequency-enhanced Spatial Multi-scale Module (PFSM) is designed, which enhances the structures of different frequency bands through Fourier transform and multi-scale convolution; the Feature-Spatial Enhancement Module (FSEM) employs a gradient-driven spatial attention mechanism to extract key regional features. Experiments on flat plate film-cooled jet schlieren data show that SFS-VAE can effectively preserve the information of the mainstream region and more accurately capture the high-gradient features of the jet region, reducing the Root Mean Square Error (RMSE) by approximately 16.9% and increasing the Peak Signal-to-Noise Ratio (PSNR) by approximately 1.6 dB. Furthermore, when integrated with a Transformer for temporal prediction, the model exhibits significantly improved stability and accuracy in forecasting flow field evolution. Overall, the model’s physical interpretability and generalization ability make it a powerful new tool for advanced flow diagnostics through the robust analysis of Schlieren sensor data.

## 1. Introduction

Schlieren imaging, as a non-invasive optical sensor system, has become an essential visual diagnostic tool for analyzing optical inhomogeneities in transparent media such as fluid flows, acoustic fields, and combustion processes. These images play a central role in modern image-based analysis pipelines, enabling researchers to extract spatial features and track flow structures [1]. Compared with particle injection or fluorescence tracking visualization techniques, schlieren technology does not require the addition of any extra substances to the fluid, allowing direct observation of refractive index gradients within, and it can more comprehensively capture the flow field. However, the raw data from schlieren sensors are often affected by noise, limited contrast, and overlapping structures, posing challenges for accurate interpretation. Consequently, the development of robust image processing and analysis methods has become a key research focus in this field [2].

Before the adoption of modern machine learning-based techniques, researchers explored a range of traditional image processing strategies to extract meaningful features from schlieren images. For example, Arnaud et al. [3] introduced divergence- and curl-based regularization in optical flow estimation for schlieren velocimetry, integrating physical constraints into motion analysis from image data. Goldhahn et al. [4] utilized background schlieren imaging to reconstruct the density field downstream of a straight blade in a wind tunnel by inferring the refractive index and density distribution from gray levels, achieving an error within 4%. Cammi et al. [5] proposed an automatic recognition method for oblique shock waves and simple wave structures based on image processing. By preprocessing and applying the Hough transform to extract linear features in the flow field, and combining physical constraints to eliminate anomalies, they successfully detected local shock wave structures in low-contrast schlieren images. Zheng et al. [6] focused on the problem of edge extraction in schlieren images, systematically reviewed and optimized the active contour model, and combined Fast Fourier Transform (FFT) with multi-scale convolution strategies to improve the stability of boundary recognition in noisy environments. These early methods marked key milestones where image processing served as the critical bridge between visualization and quantitative interpretation.

Beyond conventional feature detection and edge enhancement, mode decomposition techniques have also been widely used for analyzing schlieren image sequences. The concept of Reduced-Order Model (ROM), first introduced by Dowell et al. [7], aims to extract dominant modal structures from high-dimensional data. Among them, Proper Orthogonal Decomposition (POD) and Dynamic Mode Decomposition (DMD) are commonly used tools for dimensionality reduction and spatiotemporal pattern analysis. POD projects data onto optimal orthogonal bases, while DMD captures dynamic behaviors from time-resolved image sequences [8,9]. These techniques have been adopted in schlieren flow diagnostics—for example, Datta et al. [10] applied POD to study shock-boundary layer interactions, and Berry et al. [11] used POD and DMD to analyze nozzle flow dynamics. However, these traditional methods inherently assume linearity and often fail to capture sharp edges and local nonlinearities in raw schlieren images. Even when dozens of POD modes are used in high Reynolds number cases, the proportion of captured energy remains limited [12].

In recent years, with the rise of deep learning, Autoencoders (AE) have been regarded as a more advanced nonlinear low-dimensional feature representation method [13,14]. AE automatically extract low-dimensional features through nonlinear mapping and can effectively capture nonlinear patterns in complex flow fields. Generally, autoencoders use neural network-based encoders for feature reduction in the encoding stage and decoders for flow field reconstruction. Compared with traditional POD or DMD, autoencoders have unique advantages in capturing the nonlinear dynamic characteristics of complex flow fields, providing new opportunities for flow field mode decomposition and feature extraction. In 2019, Lee et al. [15] first introduced the Convolutional Autoencoder (CAE) into the field of fluid mechanics. Compared with linear ROM methods such as POD, the combination of autoencoder and Long Short-Term Memory (LSTM) improved the performance of flow field reconstruction and prediction, with only a marginal increase in computational cost. This basic framework has been widely used in subsequent studies. For instance, in 2021, Ahmed et al. [16] proposed a nonlinear POD method, which utilized CAE to learn the nonlinear projection of the manifold space and successfully analyzed the dynamic characteristics of periodic and chaotic flows. In 2023, Bo Zhang [17] combined CAE with a multi-head attention-based long short-term memory network to predict the unsteady flow field on an airfoil. In 2024, Xu et al. [18] developed a CAE-RNN hybrid architecture, achieving 99% dimensionality reduction in porous media turbulence research through nonlinear feature compression and combining Recurrent Neural Network (RNN) to predict temporal evolution.

With the increasing complexity of application scenarios, multi-scale architectural design has become one of the improvement directions [19,20,21]. In 2025, Lu et al. [22] proposed a CAE architecture integrating multi-scale Convolutional Block Attention Modules (CBAM), adding a term in the loss function based on the Navier–Stokes equations, so that the predicted flow respects physical conservation laws. In 2025, Teutsch et al. [23] developed SMS-CAE, which enhanced feature visualization capabilities through sparse multi-scale design, achieving a 95% modal matching rate in identifying turbulent coherent structures while maintaining 89% reconstruction accuracy, with a 26% improvement in modal identification rate compared to traditional DMD. In 2025, Beiki et al. [24] developed an attention-enhanced autoencoder combining adaptive attention mechanisms and involution layers, achieving 98.7% accuracy in the reconstruction of the transient process of flow around a cylinder, marking a 12 percent improvement over the standard CAE. Recent efforts also explored hybrid architectures, such as fully connected–convolutional–temporal networks [25,26], and parametric modeling with ResNet-based latent space mapping [27,28], for enhancing flow prediction under varying conditions.

As a generative model, the Variational Autoencoder (VAE) builds latent space representations of flow field data using a probabilistic framework, demonstrating unique advantages in nonlinear reduced-order modeling [29,30,31]. Unlike traditional autoencoders, VAE achieves nonlinear compression of flow field features by introducing distribution constraints on latent variables, providing the ability to quantify uncertainties in flow structures. In 2022, Hamidreza Eivazi [32] applied β-Variational Autoencoder (β-VAE) to LESs-simulated urban environment flow field data, achieving 87.36% energy in five modes, far exceeding 32.41% of POD. In 2024, Solera-Rico et al. [33] combined β-VAE with Transformer, achieving nearly orthogonal latent space representations in two-dimensional viscous flow prediction, with feature modes similar to POD but with significantly improved computational efficiency. In the same year, Wang et al. [34] optimized latent space decoupling by adjusting the β coefficient, reducing the turbulence energy spectrum reconstruction error to one-third of that of traditional POD. This achievement verified the adaptability of β-VAE to chemically reactive flow fields in the prediction of hydrogen fuel combustion field parameters. To meet the requirements of parametric modeling, Lee et al. [35] developed LSH-VAE, which uses a hierarchical structure and a hybrid loss function to achieve parameter interpolation prediction in fluid-solid coupling problems, reducing prediction error by 26% compared to traditional methods. Its spherical linear interpolation strategy provides a novel approach for cross-geometric topology prediction.

By leveraging probabilistic generative mechanisms, VAEs can effectively capture quasi-ordered structures and non-stationary characteristics in flows, demonstrating significant potential in flow field data reduction and feature extraction. However, when directly applied to the data from schlieren experiments, traditional VAEs struggle to focus on the key physical features dominated by refractive index gradients in schlieren images. Additionally, in dealing with complex flow features, the latent variable space representations are often ambiguous, making it difficult to effectively decouple physical modes and affecting the precise reconstruction of flow structures and temporal prediction performance. This issue is particularly pronounced in small datasets with significant noise interference in schlieren images. In contrast, linear decomposition methods like POD, although capable of providing a low-dimensional, interpretable representation of flow fields in terms of energy ranking, are limited in capturing nonlinearities, local details, and high-gradient regions. This deficiency leads to insufficient accuracy in reconstructing non-stationary and detail-rich flow structures, and the noise inherent in schlieren images further exacerbates this problem. In fact, when processing the dataset in this study, the energy captured by the first 10 modes of POD was less than 30% of the total energy.

To address challenges in processing schlieren sensor data, we propose a novel Spatial-Frequency-Scale VAE (SFS-VAE) framework based on the β-VAE architecture, which integrates frequency domain enhancement with spatial multi-scale mechanisms. The core contribution of this framework involves two specialized modules:

(1) The Progressive Frequency-enhanced Spatial Multi-scale Module (PFSM) combines discrete Fourier transform, adaptive Gaussian frequency masking, and channel-grouped spatial attention to dynamically partition the frequency spectrum and enhance the response of key frequency bands. This design improves sensitivity to fine-scale flow structures and mitigates feature loss commonly observed in traditional convolutional autoencoders during image reconstruction. (2) The Feature-Spatial Enhancement Module (FSEM) integrates gradient-driven edge enhancement, local mean-variance adaptive feature modulation, and physical priors (utilizing gradient-averaged schlieren images for guidance), enabling fine feature optimization in key regions such as jets and edge structures. By effectively decoupling and enhancing features, FSEM, together with PFSM, forms a robust VAE framework, SFS-VAE. The experimental results demonstrate substantial enhancements in low-dimensional flow reconstruction, temporal prediction accuracy, and the interpretability of the physical phenomena captured by the sensor.

## 2. Methods

Schlieren images, as grayscale representations of flow-induced refractive index gradients, carry unique characteristics that pose distinct challenges for image processing and reduced-order modeling. These images primarily capture the spatial distribution of optical inhomogeneities in transparent media, where subtle grayscale variations correspond to gradients in density or temperature. Key features include:

(1) Structural duality: This involves a relatively stable mainstream region with smooth grayscale transitions and high-gradient jet regions with sharp edges and complex vortical structures, which are critical for understanding flow dynamics. (2) Noise and contrast limitations: Raw images often suffer from low signal-to-noise ratios and uneven contrast, making it difficult to distinguish overlapping flow structures. (3) Multi-scale and multi-frequency components: Flow features span a wide range of spatial scales (from large-scale mainstream to small-scale turbulent eddies) and frequency bands (from low-frequency smooth regions to high-frequency edge perturbations). These characteristics demand specialized algorithms that can simultaneously enhance frequency-domain details, capture multi-scale spatial features, and focus on high-gradient key regions—motivating the development of the SFS-VAE proposed in this study.

### 2.1. CNN-Based β-Variational Autoencoders

To achieve an efficient reduced-order model for schlieren instantaneous flow field images, we design a neural network model architecture based on the β-VAE. The VAE is a deep generative model that introduces a probabilistic modeling framework with latent variables, enabling it to effectively learn the low-dimensional latent representation structure of image data. Compared with traditional deterministic autoencoders, VAE exhibits superior generalization ability and feature representation capabilities, performing outstandingly in capturing the distribution characteristics of complex data. The β-VAE, based on the traditional VAE, enhances the model’s constraint ability on the latent space distribution by introducing a weight factor β, thereby obtaining greater flexibility and better performance in balancing data compression and reconstruction fidelity [36].

The base network architecture in this study is based on the structure proposed by Eivazi et al. [32]. Their research added multiple convolutional layers to construct deeper encoder and decoder structures, significantly enhancing the model’s capacity for nonlinear feature extraction and structural representation. Additionally, they proposed a complete framework for latent space modeling and flow field prediction. In our implementation, the encoder network $F_{\mathrm{enc}}$ contains six convolutional blocks, each using a 3 × 3 kernel size and a stride of 2 for the convolution operation, followed by the ELU activation function to improve the nonlinear fitting ability. Through successive downsampling, the spatial resolution is gradually reduced while the number of channels increases. The resulting feature maps are flattened into a one-dimensional vector through the Flatten layer and mapped to the latent space by a fully connected layer. Therefore, the input image $I_{t}\in R^{H\times W\times C}$ will be compressed into a latent feature vector $z_{t}\in R^{d}$ after passing through the encoder. The encoder outputs two vectors $\mu_{t},log\sigma_{t}^{2}\in R^{d}$, representing the mean and logarithmic variance of the latent distribution, respectively. To achieve random sampling of latent variables and the trainability of the model, the reparameterization trick is used to generate stochastic latent variables:

(1) $$\mu_{t},\log\sigma_{t}^{2}=F_{\mathrm{enc}}\left(I_{t};\theta_{\mathrm{enc}}\right)$$

(2) $$z_{t}=\mu_{t}+\epsilon \cdot \exp\left(\frac{1}{2}\log\sigma_{t}^{2}\right),\;\epsilon ∼N\left(0,I\right)$$

The latent variable $z_{t}$ is fed into the decoder $F_{dec}$ for layer-by-layer upsampling and image reconstruction. First, it is transformed from a low-dimensional latent vector into a high-dimensional feature map using a fully connected layer followed by an unflatten operation. A six-layer deconvolutional architecture, symmetric to the encoder and employing ELU activation functions, is then used to incrementally reconstruct the image $\tilde{I_{t}}$ to its original size:

(3) $$\tilde{I_{t}}=F_{dec}\left(z_{t};\theta_{dec}\right)$$

We adopt the widely used loss function in β-VAE, which is expressed as follows:

(4) $$L_{VAE}=\underset{L_{rec}}{\underbrace{∥I_{t}-{\tilde{I}}_{t}{∥}_{2}^{2}}}+\beta \cdot \underset{L_{KL}}{\underbrace{D_{KL}\left(N\left(\mu_{t},\sigma_{t}^{2}\right)∥N\left(0,I\right)\right)}}$$

Here, the β is a tunable weight term used to balance the trade-off between reconstruction and regularization.

**Figure 1:**
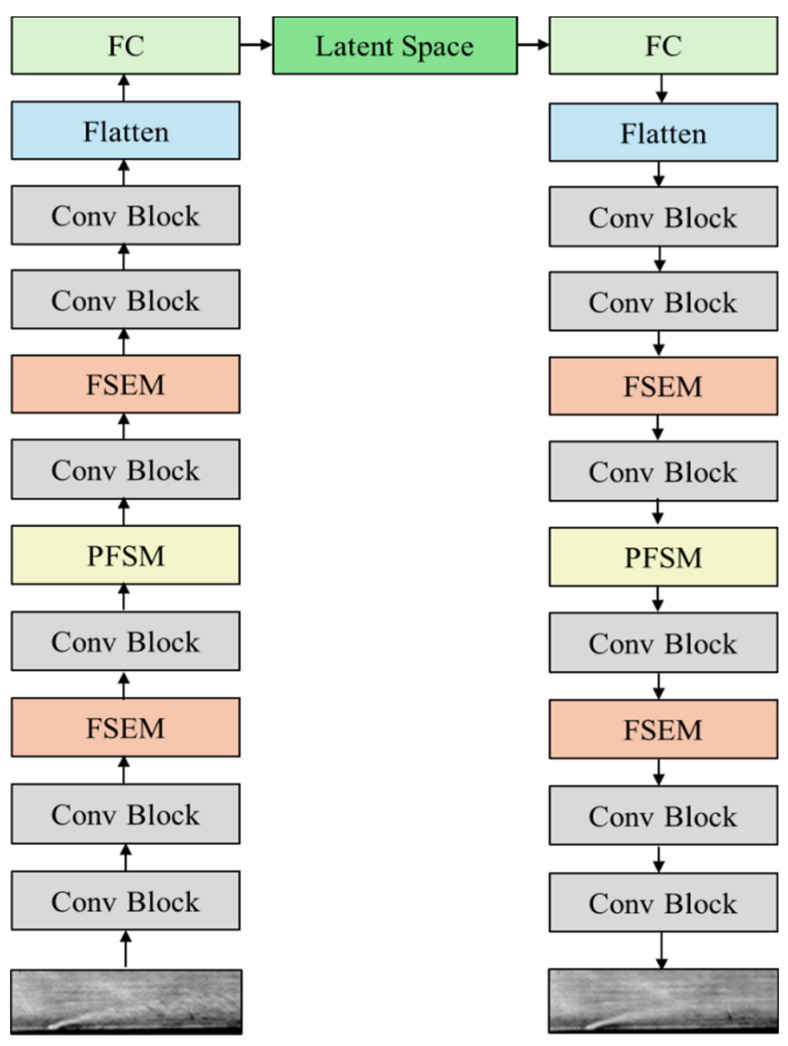
Architecture of the SFS-VAE.

To further enhance the model’s ability to represent complex instantaneous flow field structures captured by schlieren imaging, we extend and optimize the base architecture. Specifically, we introduce a Feature-Spatial Enhancement Module (FSEM) after the second and fourth convolutional layers of the encoder and a Progressive Frequency-enhanced Spatial Multi-scale Module (PFSM) after the third convolutional layer, thereby simultaneously improving the network’s feature capture capabilities in both the spatial and frequency domains. The decoder is constructed symmetrically, and the specific network architecture is illustrated in Figure 1.

### 2.2. Progressive Frequency-Enhanced Spatial Multi-Scale Module

**Figure 2:**
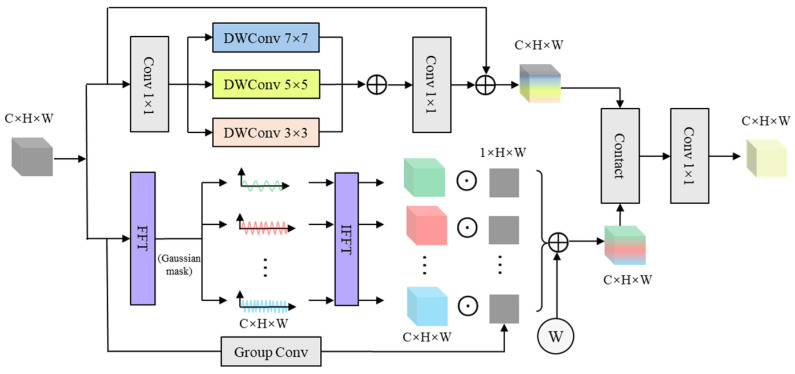
Architecture of the PFSM.

To fully exploit the diversity of flow structures in schlieren images across frequency and spatial scales, we design a Progressive Frequency-enhanced Spatial Multi-scale Module (PFSM). This module consists of two parallel branches: (1) The frequency enhancement branch, which uses the Fourier transform combined with an approximate separable Gaussian frequency mask to achieve adaptive continuous division of the spectrum and enhance the representation of flow features in the frequency domain. (2) The spatial multi-scale convolution aggregation branch, which leverages the multi-scale combination of depthwise separable convolutions to extract rich spatial scale information. Finally, the features from the two branches are concatenated and fused to comprehensively capture the spectral and spatial features of the flow structure. This improves the recognition and generalization performance for complex flow fields. The architecture of the PFSM is shown in Figure 2.

In the frequency domain enhancement branch, let the output feature map of the intermediate layer of the encoder be $X\in R^{B\times C\times H\times W}$. A two-dimensional Fast Fourier Transform is used to calculate the complex frequency spectrum representation $X_{F}=F\left(X\right)$. To construct a trainable frequency band division mechanism, instead of using the traditional discrete hard-cut segmentation mask [37], an approximate separable Gaussian frequency response function is adopted, which takes the form of:

(5) $$M_{b}\left(u,v\right)\approx M_{b,u}\left(u\right)\cdot M_{b,v}\left(v\right)=\frac{1}{1+\frac{(u-\mu_{u,b}{)}^{2}}{2\sigma_{u,b}^{2}}}\cdot \frac{1}{1+\frac{(v-\mu_{v,b}{)}^{2}}{2\sigma_{v,b}^{2}}}$$

Here, $\left(\mu_{u,b},\mu_{v,b}\right)$ represent the frequency center position, and $\sigma_{u,b},\sigma_{v,b}$ denote the frequency band width. All these parameters are trainable, enabling the model to adaptively select the most informative frequency regions during training without relying on manually defined annular frequency bands. Compared with using a complete Gaussian mask, this module significantly reduces computational costs while still maintaining the smoothness and continuity of the spectral division. The spectral mask is applied to the frequency spectrum through element-wise multiplication, and then an Inverse Fast Fourier Transform (IFFT) is performed to recover the spatial domain features corresponding to each frequency band:

(6) $$X^{\left(b\right)}=F^{-1}\left(X_{F}\cdot M_{b}\right),b=1,\ldots ,B$$

To enhance the separation ability of local disturbance structures, a shared channel group spatial attention mechanism is introduced in each frequency band branch. Specifically, the original feature map X is processed using group convolution to generate a spatial attention weight map:

(7) $$W=\sigma \left(Conv2D_{G}\left(X\right)\right)$$

Then, a channel-wise broadcast element-wise multiplication is performed between the spatial weight map and the original frequency band feature map to enhance the regions that are sensitive to flow structures. This results in the modulated response:

(8) $${\hat{X}}^{\left(b\right)}=W⊙X^{\left(b\right)}$$

Subsequently, through the weighted fusion of the frequency bands, the spatial feature expressions of all frequency band responses are integrated to form the final frequency domain enhanced feature output:

(9) $$X_{freq-out}=\sum_{b=1}^{B}\;\alpha_{b}{\hat{X}}^{\left(b\right)}$$

Here, $\alpha_{b}$ is a learnable fusion weight coefficient, allowing the model to adaptively determine the contribution of each frequency band to the final feature representation.

To reflect the diverse spatial-scale characteristics of flow structures in schlieren images, a multi-scale convolutional aggregation branch in the spatial domain is introduced. This branch also takes the output feature map $X\in R^{B\times C\times H\times W}$ from the intermediate layer of the encoder as input. It first performs a 1 × 1 convolution for dimensionality transformation to obtain the intermediate feature map $X_{pre}\in R^{B\times C^{\prime }\times H\times W}$. Then, Depthwise separable convolutions (DWConv) with three different-sized kernels (3 × 3, 5 × 5, 7 × 7) [38] are used to extract spatial features at different scales:

(10) $$Y_{s}={DWConv}_{k_{s}}\left(X_{pre}\right),s=1,2,3$$

The feature maps at each scale are then summed and merged through a pointwise convolution (1 × 1). Finally, a residual connection is introduced to stabilize training and enhance the feature representation capability, resulting in the output of multi-scale aggregated features in the spatial domain:

(11) $$X_{\mathrm{spatial}\;\mathrm{out}}=X+PWConv\left(\sum_{s=1}^{3}\;Y_{s}\right)$$

After the feature extraction of the two branches is completed, the frequency domain and spatial domain features are concatenated along the channel dimension, then fused and dimensionally reduced through pointwise convolution (1 × 1 convolution) to obtain the output feature after fusion.

(12) $$X_{PFSM}={\mathrm{Conv}}_{1\times 1}\left(\left[X_{freq-out},X_{\mathrm{spatial}-\mathrm{out}}\right]\right)$$

This two-branch design captures and combines features from schlieren images in both frequency and spatial domains, making the reconstruction more accurate.

### 2.3. Feature-Spatial Enhancement Module

**Figure 3:**
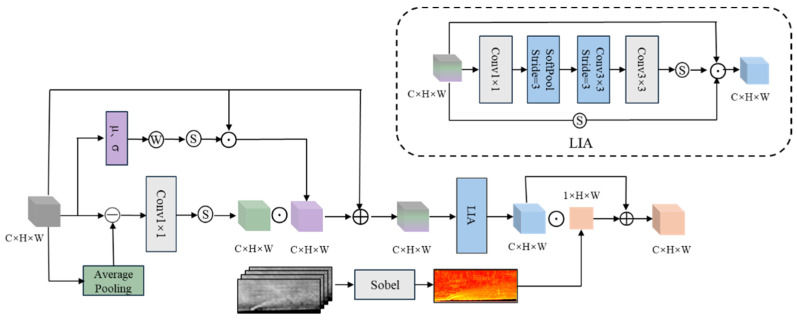
Architecture of the FSEM.

To more accurately capture the local details and physical structure contours of the jet region, we design a Feature-Spatial Enhancement Module (FSEM), which improves local details by using edge-based weights, applying attention to important areas, and introduces the spatial fusion of external physical priors. The specific architecture is shown in Figure 3.

Let the intermediate layer feature map of the encoder be denoted as $X\in R^{B\times C\times H\times W}$. To highlight the details and edge structures of key flow regions such as vortices, shear layers, and boundary layers in the schlieren image, we introduce an edge enhancement mechanism based on residual gradient features. The input features are subjected to a 3 × 3 average pooling operation to obtain the locally smoothed mean feature map, denoted as $P\left(X\right)$, and then a residual structure is used to extract fine-grained edge feature responses [39]:

(13) $$E=X-P\left(X\right)$$

This residual operation can effectively capture subtle boundary variations in the schlieren image and suppress the interference of large-scale smooth regions. The edge response E is processed through a 1 × 1 convolution, Batch Normalization, and Sigmoid activation to generate a spatial edge weight map:

(14) $$W_{edge}=\sigma_{sigmoid}\left(BN\left({Conv}_{1\times 1}\left(E\right)\right)\right)$$

We further propose a local mean-variance feature enhancement method, which utilizes the spatial mean and variance of feature maps and employs a small number of training parameters to achieve adaptive spatial feature modulation:

(15) $$X_{enhanced}=X⊙\sigma_{sigmoid}\left(\alpha {\left(X-\mu \right)}^{2}+\beta \sigma \right)$$

Here, μ, σ represent the spatial mean and standard deviation of the feature map, respectively, and α, β are learnable parameters. Subsequently, the enhanced spatial features will be combined with the edge weights:

(16) $$X^{\prime }=W_{edge}⊙X_{enhanced}+X$$

To enhance the spatial representation ability of local structures in images, the previously enhanced features are further processed by the Localized Soft Pooling Attention (LIA) mechanism, which was originally proposed by Wang et al. in the field of image super-resolution [40]. First, a 1 × 1 convolution is applied to the feature map $X^{\prime }$ to reduce the channel dimension. Then, a 3 × 3 SoftPooling is used along with the exponentially weighted aggregate to obtain the neighborhood information:

(17) $$Y_{i,j}=\frac{\sum_{\left(m,n\right)\in N_{i,j}}\;e^{X_{m,n}^{\prime }}X_{m,n}^{\prime }}{\sum_{\left(m,n\right)\in N_{i,j}}\;e^{X_{m,n}^{\prime }}}$$

Here, $N_{i,j}$ represents the local neighborhood window area centered at position $\left(i,j\right)$).

Subsequently, a 3 × 3 convolution with a stride of 2, followed by a Sigmoid function, is utilized to obtain the local spatial weight map $W_{local}$. To further differentiate between significant regions and weak disturbances, a simple gating mechanism $G=\sigma \left(X^{\prime }\left[:,0,:,:\right]\right)$ is introduced. The final spatial enhanced feature representation is expressed as:

(18) $$X^{\prime \prime }=X^{\prime }⊙W_{local}⊙G+X^{\prime }$$

To more effectively integrate physical prior information, the module also introduces spatial attention guidance derived from external physical fields. Considering the characteristic that the gray value of the schlieren image correlates with the density gradient, we utilize the average schlieren gradient map $\bar{J}$, which is obtained through external preprocessing. This map is bilinearly interpolated to match the spatial dimensions of the early -stage features, and is fused via an additional spatial attention mechanism:

(19) $$X_{FSEM}=X^{\prime \prime }+X^{\prime \prime }⊙\bar{J}$$

This fusion strategy explicitly injects flow-specific priors into the feature extraction process, thereby enhancing the network’s ability to capture fine flow details and edge structures. As a result, the model’s reconstruction accuracy and generalization performance in jet regions are significantly improved.

## 3. Construction of Datasets

In the design of modern high-bypass gas turbines, increasing demands on blade material temperature resistance have been imposed to improve overall efficiency. Consequently, exploring efficient cooling technologies to reduce the base temperature of turbine blades is essential. Film cooling, as an efficient cooling method in aeroengines, has been widely applied in turbine design. Studying the flow and cooling mechanism of film cooling jets under different jet parameters helps to elucidate their influence on gas-thermal coupling and cooling effect.

The training data employed in this study were obtained from the film cooling jet experiments conducted in a suction-type flat plate wind tunnel at Nanjing University of Aeronautics and Astronautics. The flat plate jet experiment system mainly consists of the main flow system and the secondary flow system. In actual turbines, film cooling jets are generally in the form of a combination of hot gas in the main flow and cold gas in the jet. However, due to the limited experimental conditions in university settings, the current experiment adopts cold gas as the main flow and hot gas in the jet. Although this setup may cause a decrease in jet density and an increase in jet velocity under the same blowing ratio, affecting the wall-attachment phenomenon, it still effectively captures the essential characteristics of gas-thermal coupling in film cooling scenarios.

**Figure 4:**
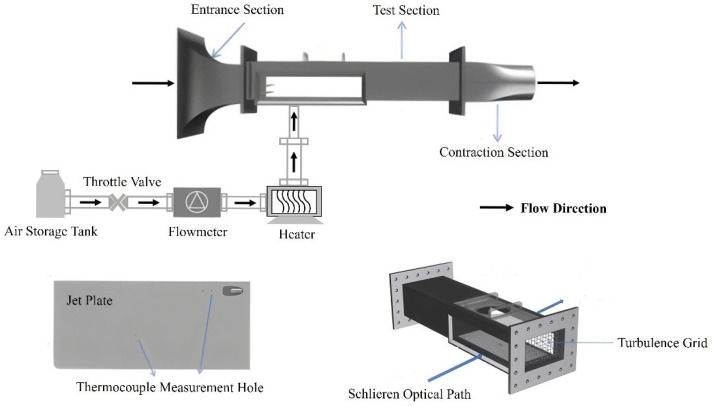
Structural diagram of the flat plate jet experiment system.

The structure of the flat plate jet experiment system is shown in Figure 4, and the suction-type flat plate wind tunnel main modules include:

- Square inlet contraction section: The inlet is equipped with fine grids, which can effectively intercept dust, reduce the probability of equipment failure, and minimize the interference from the external environment. The mainstream turbulence intensity is controlled within 0.8% to 1%.
- Test section: The overall size of the wind tunnel test section is moderate. A flat plate with holes is installed at the bottom as the test piece, with the holes connected to a series of devices for generating jet flows. Sensors are arranged on the plate to monitor turbulence intensity and surface temperature in real time. For schlieren imaging, optical glass panels are installed on both sides of the test section, and a sealed plate is installed on the top, with reserved holes for installing hot-wire probes and observation windows.
- Square-to-round transition and suction pipe: These components ensure stable fluid inflow and outflow, forming a closed circulation loop with the compressor.

In this experiment, the main adjustable jet parameters include the jet blowing ratio (M) and the jet temperature ratio (Tr), defined as:

(20) $$M=\frac{\rho_{jet}*u_{jet}}{\rho_{m}*u_{m}}=\frac{\frac{G_{jet}}{A_{jet}}}{\rho_{m}*u_{m}}$$

(21) $$Tr=\frac{T_{jet}}{T_{m}^{*}}$$

where $\rho_{jet}$, $u_{jet}$, $T_{jet}$, $G_{jet}$ represent the density, velocity, temperature and mass flow rate of the jet, respectively, and $A_{jet}\;$ is the cross-sectional area of the jet orifice. $\rho_{m}$, $u_{m}$, $T_{m}^{*}$ correspond to the density, velocity and total temperature of the mainstream in the test section. The mass flow rate of the jet is controlled and adjusted by a fixed orifice and a flowmeter, while the jet temperature is adjusted by a heater. To facilitate precise control and real-time monitoring, a compressor with a maximum working pressure of 0.8 MPa is used as the gas source, in combination with a throttle valve and a flowmeter to achieve continuous regulation.

**Figure 5:**
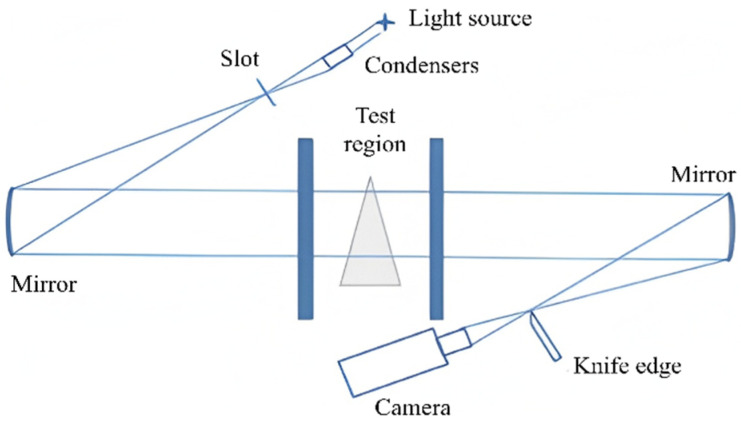
Optical path of the Z-shaped schlieren system.

**Figure 6:**
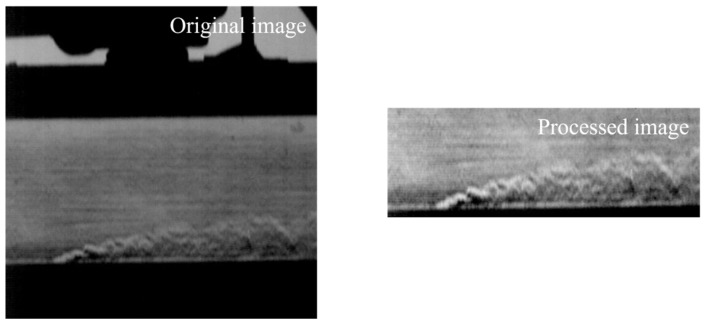
Schlieren images of the gas film jet experiment.

Data acquisition was carried out using a conventional “Z”-shaped schlieren system configuration [41], as shown in Figure 5. The system utilized a Phantom VEO 710 L high-speed camera (CMOS sensor, pixel size 20 μm, maximum resolution 800 × 1280) to capture transient flow field images. Under two mainstream Mach numbers (0.1 and 0.35), a total of 20 operating conditions were tested with jet blowing ratios M = 0.5–2.5 and jet temperature ratios Tr = 1.2 or 1.3. During acquisition, the wind tunnel flow was stabilized by controlling stagnation pressure, temperature, and humidity, and optical path vibration was minimized through mechanical isolation. The “Z” optical setup was meticulously aligned, with knife-edge position optimized to enhance density-gradient contras. The high-speed camera was set at 10,204 Hz and 512 × 512 resolution, providing sufficient temporal and spatial fidelity for the proposed SFS-VAE method. Raw schlieren frames were processed via Gaussian filtering to suppress high-frequency noise and contrast enhancement to highlight shear layers and vortex structures, followed by cropping to 320 × 128 pixels to remove non-flowfield elements and match model input requirements. These measures ensured high signal-to-noise ratio, sharpness, and contrast in the processed data, supporting accurate feature decomposition, reconstruction, and prediction in later analyses. Figure 6 presents both the raw schlieren image and the corresponding processed image from one experiment. In this case, the original image quality was sufficiently high that Gaussian filtering was applied with a 1 × 1 kernel (effectively no smoothing), and the contrast was enhanced by a linear scaling factor of 1.6 to improve the visibility of shear layers and vortex boundaries. In the lower part of the flow field, the jet structure is clearly visible, while weaker transverse mainstream features appear in the background.

## 4. Results and Discussion

This section evaluates the performance of the proposed reduced-order model in the reconstruction and prediction of the flow field from schlieren image sequences. The study utilized the dataset from the gas film jet schlieren experiment with a Mach number of 0.1, a temperature ratio of 1.3, and a blowing ratio of 2.5, comprising a total of 3120 images. In the experiment, the latent space dimension was fixed at r = 10 to balance reconstruction accuracy and computational efficiency. The dataset was divided into a training set (80%) and a test set (20%) based on the time sequence. The results of the test set were inferred solely through the trained model to ensure an objective assessment of the model’s generalization ability.

The implementation and training process of the model were implemented in PyTorch 2.3.0 to ensure code consistency and experimental reproducibility. During the experiment, the model was trained using an NVIDIA GeForce RTX 4070 (12 GB), with the Adam optimizer and a dynamic one-cycle learning rate scheduling strategy. The initial learning rate was set at 1 × 10^−4^, reaching a peak of 5 × 10^−4^ in the first 20% of the training cycle, and then decreased progressively to 1 × 10^−5^ by the end of the training. The entire training process lasted for 400 epochs, with a batch size of 256. An early stopping strategy was implemented; if the validation loss did not show a decreasing trend for 50 consecutive epochs, the training was prematurely terminated. Table 1 summarizes the model architecture, including layer types and output shapes. The hyperparameter β was set to 0.01, and a discussion on β will be provided in Section 5.

### 4.1. Evaluation of Reconstruction Performance

We aim to evaluate the performance of the PFSM, the FSEM, and the integrated SFS-VAE model. In the reduced-order reconstruction of schlieren image flow fields, we conduct a systematic analysis of the original model, ablation models, and the final model. This evaluation includes both visual comparisons of image reconstruction quality and quantitative metrics. The objective is to verify the contribution of each module to the model’s reconstruction capability, especially in accurate modeling of high-density gradient regions such as jets. To quantify the model’s performance in schlieren image reconstruction process, three standard image similarity metrics are employed: Root Mean Square Error (RMSE), Peak Signal-to-Noise Ratio (PSNR), and Pearson Correlation Coefficient (PCC). Their definitions are as follows:

- PSNR measures the fidelity of the reconstructed image, expressed in decibels (dB), and is defined as:

(22) $$PSNR=10\cdot \log_{10}\left(\frac{L^{2}}{MSE}\right)\#$$

where L represents the maximum possible pixel value, and MSE is the mean square error.

- RMSE quantifies the average error between the predicted image and the reference image and is defined as:

(23) $$RMSE=\sqrt{\frac{1}{N}\sum_{i=1}^{N}\;(x_{i}-{\hat{x}}_{i}{)}^{2}}$$

- PCC evaluates the linear correlation between the predicted and reference images, and is defined as:

(24) $$PCC=\frac{\sum_{i=1}^{N}\;(x_{i}-\bar{x})({\hat{x}}_{i}-\bar{\hat{x}})}{\sqrt{\sum_{i=1}^{N}\;(x_{i}-\bar{x}{)}^{2}}\cdot \sqrt{\sum_{i=1}^{N}\;({\hat{x}}_{i}-\bar{\hat{x}}{)}^{2}}}$$

Here, $x_{i}$ and ${\hat{x}}_{i}$ represent the i pixel values of the reference and reconstructed images, respectively; $\bar{x}$ and $\bar{\hat{x}}$ denote the mean values of the corresponding images; and N is the total number of pixels.

**Figure 7:**
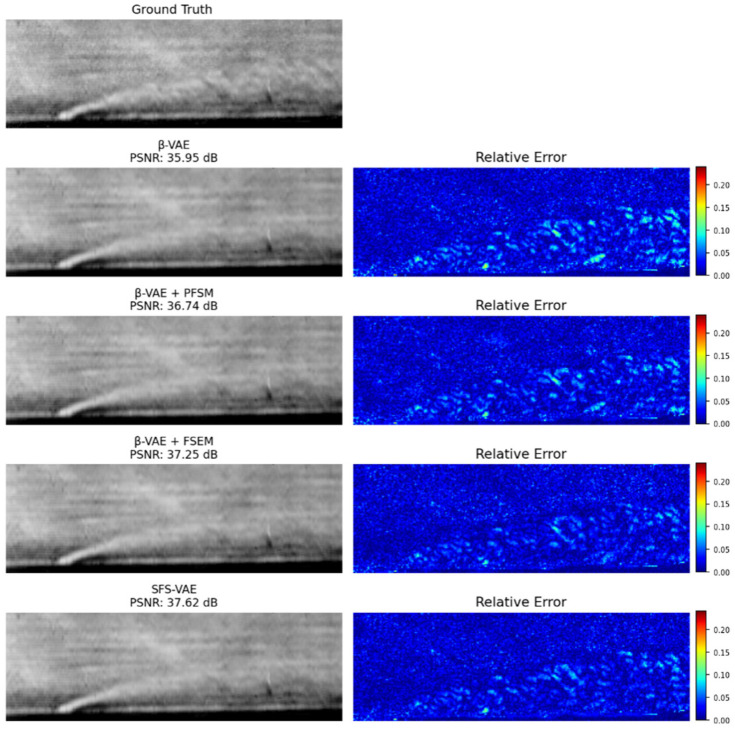
Reconstruction and Error Maps at t=10. The relative error maps are computed by calculating the point-wise relative error between the original and reconstructed snapshots (“β-VAE + PFSM” refers to the ablation model obtained by removing all FSEM from the SFS-VAE, retaining only the PFSM integrated with the original β-VAE structure. “β-VAE + FSEM” is similarly defined by removing the PFSM. These abbreviations are consistently used throughout this paper unless otherwise stated).

Figure 7 visually compares the reconstructed images of each model at t=10 in the test set with the original image. All models exhibit satisfactory reconstruction performance in the mainstream area that occupies the majority of the image, thus achieving high PSNR values. However, the relative error map reveals that the standard β-VAE model produces noticeable edge blurring artifacts in high-disturbance areas, particularly along the upper and lower boundaries of the jet and within the wake region. In these areas, the model struggles to capture sharp structural variations and grayscale transitions. In contrast, the model incorporating the PFSM shows improved preservation of flow structure boundaries, as evidenced by the clarity of vortex structures and the capture of structural details in the jet and mainstream. This indicates that the PFSM can effectively enhance the model’s perception of local frequency features and improve the clarity of structure restoration. Furthermore, after adding the FSEM, the model exhibits a significantly increased focus on the jet area, as shown by the stronger response in high-density gradient regions in the schlieren image. Notably, the overall reconstruction effect of the lower edge and wake is notably improved, which is highly consistent with the high-gradient regions in the gradient prior map. This suggests that the gradient prior mechanism embedded in the FSEM effectively guides the network’s attention to physically meaningful regions, enhancing the reconstruction of high-density disturbance structures. However, due to the presence of the prior mechanism, the model’s reconstruction error in the mainstream, an area with relatively low significance, is slightly higher than that of the PFSM ablation model and contains more noise-like artifacts. In comparison, the SFS-VAE model integrates the advantages of both models, effectively maintaining the global structure while capturing richer local details, and achieves the highest PSNR of 37.62 dB.

**Figure 8:**
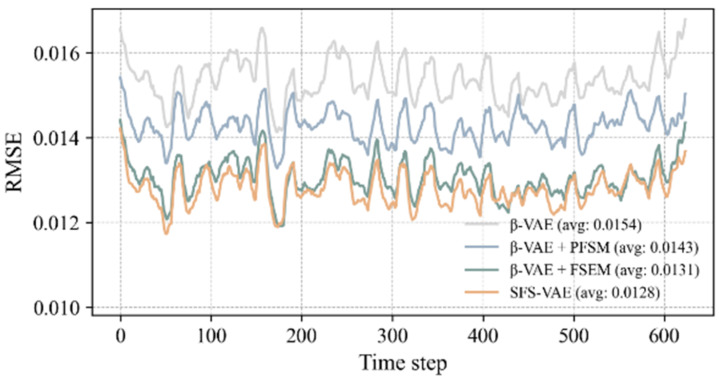
Time-series curve of RMSE.

**Figure 9:**
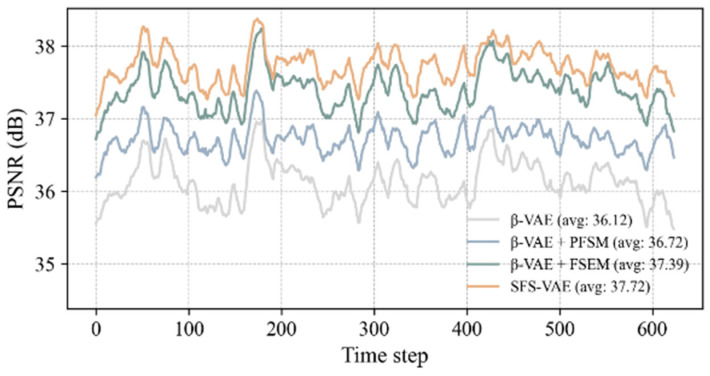
Time-series of the PSNR.

To further quantify the reconstruction capabilities of each model, we statistically analyzed the reconstruction error and image similarity metrics on the time series of the entire test set, as shown in Figure 8 and Figure 9. Firstly, the RMSE was calculated to measure the overall reconstruction deviation. The experimental results indicated that the average RMSE of the complete model at all times was 0.0128, the lowest among all models, demonstrating superior reconstruction performance. Secondly, the PSNR also showed that the complete model had a significant advantage in signal-to-noise control, with its average PSNR substantially higher than that of the ablation models and the standard model. Compared to the original β-VAE, the RMSE was reduced by approximately 16.9%, and the PSNR was increased by 1.6 dB.

Overall, the reconstruction performance is strong at earlier time steps, but as time progresses, the metrics exhibit a fluctuating trend rather than a continuous decline. We attribute this behavior to two primary reasons: (1) the reconstruction quality of the mainstream region significantly influences the overall metrics, and since this region has maintained high-quality reconstruction fidelity, fluctuations in the metrics are mainly caused by variations in the jet region; (2) snapshot data suggest that the jet in this experiment exhibits quasi-periodic behavior, allowing the model to apply flow features learned from the training set to unseen patterns in the test set.

**Figure 10:**
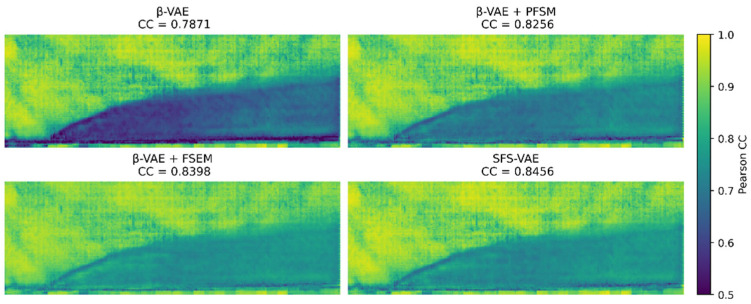
Average mapping of the PCC.

In the comparative analysis shown in Figure 10, it can be clearly observed that all models perform well in the mainstream region. However, the standard model shows relatively low correlation in the initial zone of the jet structure, the lower boundary, and the wake region, indicating an inaccurate capture of the spatiotemporal evolution trends in these key physical areas, particularly with pronounced correlation discontinuities at the initial zone and edge structures. The PFSM model improves the reconstruction performance in the mainstream area and the initial zone of the jet, significantly mitigating the phenomenon of correlation breaks in the latter. Meanwhile, the FSEM model demonstrates excellent overall performance in reconstructing the jet region, with noticeable improvements in edge continuity. In contrast, the complete model exhibits consistently higher correlation values that are more spatially concentrated and temporally continuous, suggesting superior robustness and expressive capacity in physical structure extraction and temporal dynamics modeling. This outcome further confirms the contribution of PFSM in enhancing the consistency of local edge structures in the frequency domain, as well as the improvements in modeling accuracy of highly perturbed regions brought about by the gradient-driven spatial attention mechanism in FSEM.

Based on the statistical analysis of multiple quantitative metrics, the complete model consistently outperforms both the original and ablation models in terms of RMSE, PSNR, PCC, and other indicators. This demonstrates the effectiveness of the proposed modules in reconstructing Schlieren flow fields, particularly in enhancing the fidelity of jet structure boundaries and regions characterized by high-gradient disturbances.

### 4.2. Analysis of Latent Space Modes

To further investigate the modal decomposition ability of models for schlieren flow field data, this study compares the differences in modal extraction and energy distribution across various models. Previous works have consistently employed the analysis method proposed by Eivazi [29,30,31]. The core idea is to use the decoder to convert each individually activated latent variable in the latent space into a spatial mode. Specifically, for a trained model, the i-th component of the latent variable vector r obtained through the encoder is retained while the other components are set to 0 to obtain a modal vector ${\hat{r}}_{i}$, and then it is sent to the decoder for reconstruction to obtain the representation graph ${\tilde{x}}_{i}$ of the i-th mode. By comparing the decoded mode with the actual data and calculating the percentage of the total energy $E_{k}^{i}$ occupied by the reconstructed image of this mode, the importance of the i-th mode is quantified:

(25) $$E_{k}^{i}=\left(1-\left\langle \frac{\sum_{j=1}^{n}\;{\left(u-\tilde{u}\right)}^{2}}{\sum_{j=1}^{n}\;u^{2}}\right\rangle \right)\times 100$$

Here, $\left\langle \cdot \right\rangle$ represents the ensemble average over time, u) and $\tilde{u}$ denote the reference gray value and the reconstructed gray value, respectively, and n represents the total number of pixels in the image. By statistically analyzing the contribution of each mode in the reconstruction, the first mode $E_{k}^{1}$, which carries the highest energy, can be identified. The second mode is defined as the one that, when added to the first mode, contributes the most additional energy to the reconstruction.

Meanwhile, to evaluate the orthogonality between the latent vectors, the correlation matrix R is calculated as:

(26) $$R=(R_{ij}{)}_{d\times d}$$

(27) $$R_{ii}=1\;and{\;R}_{ij}=\frac{C_{ij}}{\sqrt{C_{ii}C_{jj}}}$$

Here, $C_{ij}$ represents the element in the i -th row and j-th column of the covariance C, and d is the dimension of the latent space. When the variables are completely uncorrelated, $R_{ij}=0$, and when they are completely correlated, $R_{ij}=1$. Additionally, the determinant of this correlation matrix, ${det}_{R}$, is calculated and scaled by a factor of 100 to serve as a metric for evaluating the degree of independence among the latent variables.

**Figure 11:**
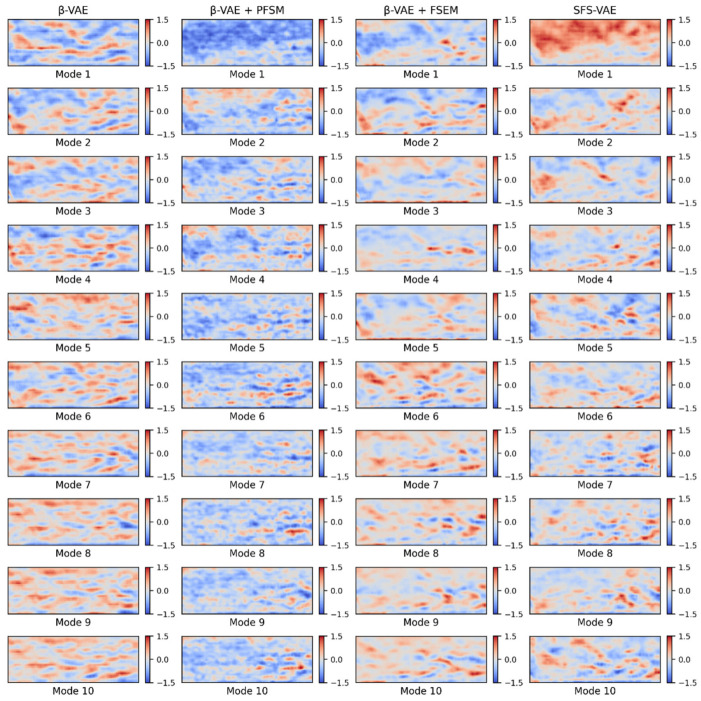
Modes of β-VAE, β-VAE + PFSM, β-VAE + FSEM, and SFS-VAE (Red areas indicate positive deviations—an increase in the normalized pixel intensity relative to the mean, corresponding to regions of higher refractive index gradient in the Schlieren image; blue areas indicate negative deviations—a decrease in the normalized pixel intensity, corresponding to regions of lower refractive index gradient).

Based on the above principles, this study compares the performance of β-VAE, the final model SFS-VAE, and two ablation models. Figure 11 shows the top 10 modes calculated by the four models, and the images of each mode visually reflect the corresponding physical feature distribution. The modes generated by β-VAE typically contain relatively broad global information but exhibit weak differentiation between modes and low physical interpretability. Modes 1–5 appear to aim at capturing quasi-ordered flow structures in the jet region, typically manifested as red-blue alternating patterns within the jet shear layer. However, these modes also contain many features originating from the mainstream flow, introducing predominantly transverse orientations that are inconsistent with the jet’s direction. This mixing reduces the clarity and physical interpretability of the jet-related features. Crucially, the model fails to capture the expected downstream evolution: instead of presenting the desired breakdown into smaller, densely spaced structures in higher-order modes, the influence of mainstream components becomes even more pronounced, further obscuring physically meaningful jet information and preventing the extraction of valuable flow features. These observations suggest that the original model is strongly influenced by noise and background disturbances during feature separation. From the perspective of energy ranking, the mainstream area occupies a large portion of the image and has a substantial impact on the image reconstruction index. Hence, the modes corresponding to the mainstream region should appear earlier. However, in the β-VAE results, a distinct mode representing the mainstream region is not clearly observed; instead, it coexists with the quasi-ordered jet structures in higher modes. In contrast, for the ablation model β-VAE + PFSM, the energy of the first mode is well concentrated in the mainstream area, despite being classified into the blue region. And most of the subsequent modes are related to the quasi-ordered structures of the jet, with relatively small quasi-ordered structure sizes. This indicates that PFSM enhances the model’s ability to capture high-frequency and small-scale flow structures through frequency enhancement and multi-scale convolution. However, the problem that remains is that the mode distinction is still low, and the correlation between some modes is slightly high, as shown in the related matrix graph in the following text. Physically, high inter-mode correlation suggests redundancy in representing similar flow phenomena, reducing the decomposition’s effectiveness in isolating distinct coherent structures. For the other ablation model β-VAE + FSEM, its ability to decouple the jet region is substantially improved, with all 10 modes showing varying degrees of jet-region decomposition. Higher-order modes also capture finer quasi-ordered structures, appearing as more densely spaced red-blue alternating patterns in the jet region. The final model, SFS-VAE, inherits the advantages of FSEM and PFSM. The first mode primarily captures the mainstream region, while the subsequent modes more precisely identify the high-gradient regions within the jet structure. These modes exhibit clearer and more physically interpretable flow structures. Specifically, several modes preserve the wave-like patterns along the shear layer seen in the original modal decomposition. Additional modes capture the downstream breakdown of vortices into smaller, more densely spaced structures, reflecting the transition to higher-frequency modes, this progression is consistent with the energy cascade in turbulent flows, where large-scale vortices transfer kinetic energy to progressively smaller structures downstream.

**Figure 12:**
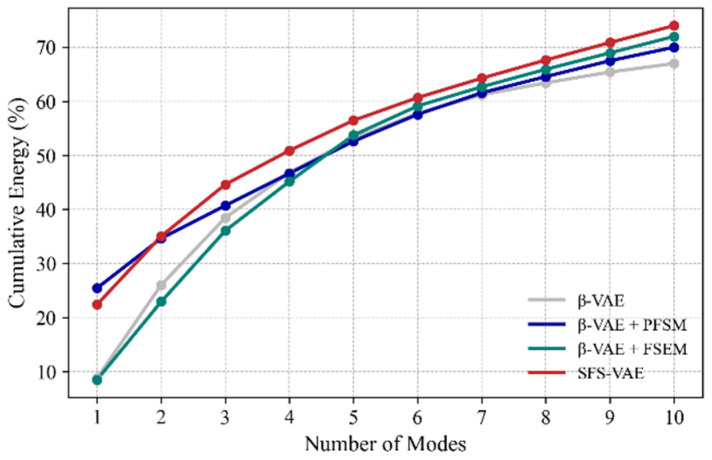
Energy accumulation curves of the four models.

Figure 12 illustrates the cumulative energy curves of the top 10 modes for all four models. Upon comparison, it is evident that the SFS-VAE model achieves the highest cumulative energy within the first 10 modes, reaching approximately 73.8%. In contrast, β-VAE captures only 67.2%, indicating lower efficiency in data compression and feature extraction. The cumulative energies of the two ablation models fall in between, but both are still inferior to that of SFS-VAE. In addition, both energy concentration and mode ranking stability are reduced in the ablation models. Furthermore, since both the ablation model β-VAE + PFSM and SFS-VAE capture the mainstream flow structure in their first mode, the energy content of the first mode is significantly higher than in the other two models.

**Figure 13:**
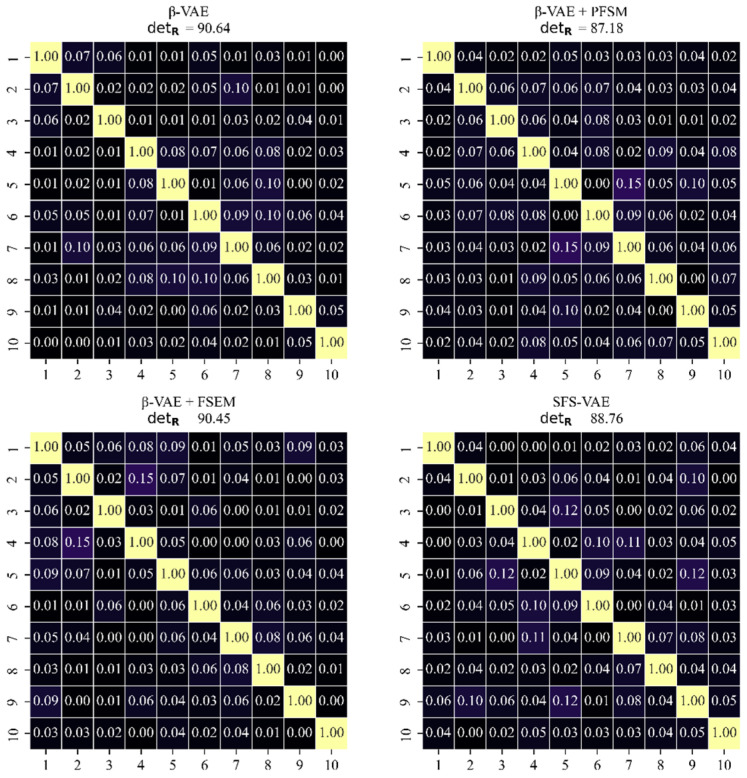
The correlation matrices for the four models.

Figure 13 presents the correlation matrices of four models. Among them, the model with the highest ${det}_{R}$ is the original β-VAE, reaching 90.64, while the lowest is β-VAE + PFSM at 87.18. The ${det}_{R}$ of the β-VAE + FSEM model shows minimal change, and the complete model SFS-VAE has a ${det}_{R}$ of 88.76. It is evident that the introduction of the PFSM enhances the inter-mode correlations. Through experimental analysis, we attribute this effect to the spatial-domain multi-scale convolution aggregation branch, which integrates three convolution kernels of varying sizes to perform multi-scale fusion. While this architecture allows the model to better capture the flow field’s multi-scale structures, it also intensifies inter-feature interactions. Experimental results further reveal that increasing the number of convolution kernel types leads to a further reduction in ${det}_{R}$ due to greater overlap among the fused multi-scale features. This overlap introduces redundancy into otherwise independent feature spaces, thereby compromising the orthogonality among modes. Consequently, we ultimately retain three convolution kernels in the spatial-domain multi-scale convolution aggregation branch. Although this slightly reduces orthogonality, the trade-off remains within an acceptable range.

### 4.3. Evaluation of Time-Series Prediction Performance

To achieve accurate temporal prediction of latent vector evolution in the flow field, this study adopts a modeling approach based on the Transformer architecture. Specifically, the latent vector sequence extracted from the VAE framework serves as the temporal input. After processing by the Transformer-based temporal prediction model, the predicted schlieren flow field images are reconstructed via the decoder of SFS-VAE. In the experiment, Long Short-Term Memory (LSTM) is employed as a baseline method for temporal prediction [42]. As a Recurrent Neural Network (RNN) variant with three gates (input, forget, output) and a cell state, it addresses traditional RNNs’ long-term dependency issue to model sequential data effectively. Three variants of the Transformer model are constructed for comparative evaluation. The core architecture follows the seq2one Transformer design proposed by Wang [34]. The embedding module is implemented as a Time-Space Embedding layer, designed to encode positional information in the temporal sequence. It also enriches spatiotemporal representations via diverse pooling operations. This Time-Space Embedding is adopted in our model, while the Transformer backbone follows the current mainstream Pre-Norm architecture, which has been widely adopted in models such as BERT and the Vision Transformer (ViT). Compared with original Post-Norm, Pre-Norm provides better training stability and convergence in deep networks [43,44].

**Figure 14:**
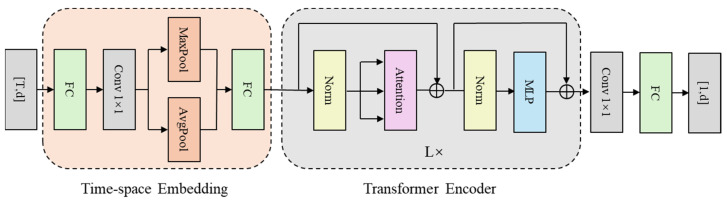
Architecture diagram of the transformer time-series prediction model.

Therefore, the latent vectors with a size dimension of d and a time dimension of T will eventually pass through the Time-space Embedding and Transformer modules, and be modulated by a one-dimensional convolutional layer and a fully connected layer to output the future latent vectors with a dimension of d. The overall architecture is depicted in Figure 14.

The traditional Transformer module employs the classic multi-head attention mechanism to model global dependencies through standard Q-K-V computations. To explore the impact of different attention mechanisms on time-series prediction performance, we replaced the multi-head attention module in the Transformer with De-stationary Attention (DSAttention) and ProbSparse Self-Attention (ProbAttention), as proposed in recent time-series prediction models [45,46]. DSAttention enhances computational efficiency and sensitivity to critical temporal features via dynamic sparsification strategies, while ProbAttention mitigates the influence of redundant information through a probabilistic sampling mechanism, demonstrating superior stability in long-term sequence modeling.

Regarding training settings, all models follow a unified time-series prediction protocol: 80% of the data is used for training and 20% for testing, consistent with the data split used in the preceding VAE model training. Specifically, the latent variables $r\left(t_{\mathrm{train}}\right)$ generated from the training set are used for model training, while the latent vectors $r\left(t_{\mathrm{test}}\right)$ obtained from the test set are used for evaluation. This ensures that all models are trained and validated under identical data conditions, thereby maintaining the comparability of results. During training, a time-delay window of 64 steps is used to predict the latent variable for the subsequent time step. The training objective is to minimize the Mean Squared Error (MSE) between the predicted and actual sequences. Appropriate regularization strategies are introduced to prevent overfitting. In the experiments, the Transformer model comprises four Transformer blocks, each equipped with four attention heads, and the MLP layer has a dimensionality of 128. The LSTM model adopts a stacked two-layer architecture with a hidden state size of 256.

**Figure 15:**
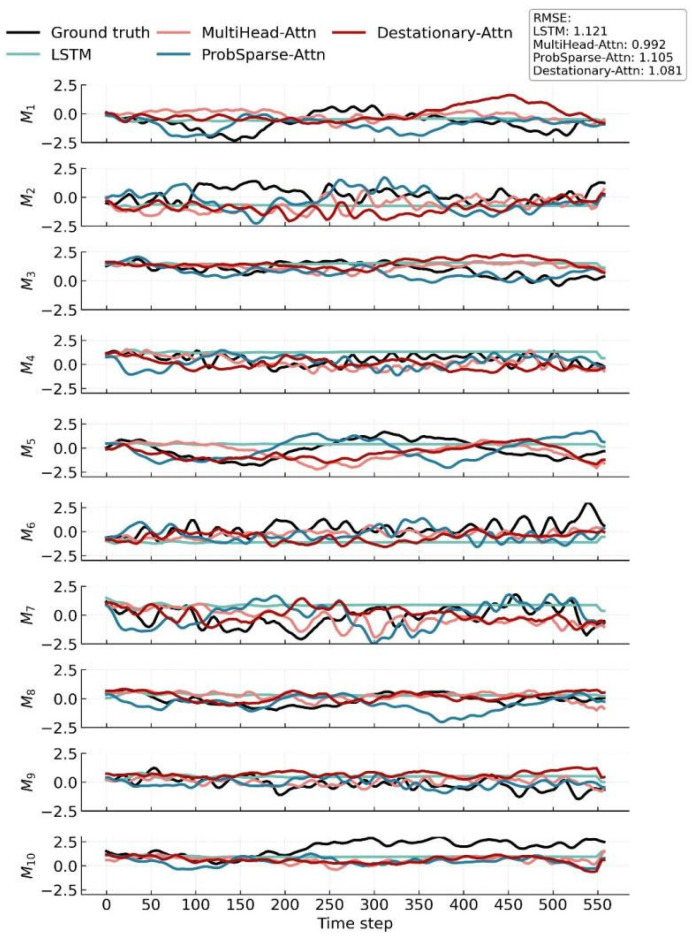
Results of various time-series prediction models in latent vector prediction.

Figure 15 compares the predicted and true time series for the 10 components of the latent vector, corresponding to the 10 spatial modes, using various time-series prediction models, where each subplot illustrates the temporal evolution of one latent component. It can be clearly seen that the LSTM-based prediction can capture the frequency changes in the latent space well before 50-time steps, but it shows a significant smoothing problem in long-term predictions, resulting in a large prediction deviation. Transformer architecture with standard multi-head attention performs the best in overall prediction. Its advantage lies in the global computation mechanism that can fully capture global context information, especially for the time series features in non-mutating regions, making the predicted curve highly consistent with the true value overall, with the RMSE being the lowest error among the four models at 0.992. However, it may have errors in more dynamic latent vectors such as 5 and 10, and also at mutation points in multi-mutating latent vectors such as 1, 2, and 6. The Transformer architecture with De-stationary Attention (DSAttention) shows a similar trend to multi-head attention in prediction, but lags slightly behind multi-head attention in some modes such as 7 and 9. Therefore, the final result is weaker than that of multi-head attention. We believe this might be because dynamic sparsification requires setting a threshold or judging key positions based on local activation values. If the threshold is not sensitive enough in some cases, it may ignore some edge information or respond insufficiently to continuous mutation information, leading to a decrease in prediction accuracy and making the overall result weaker than the traditional multi-head attention. Transformer with ProbSparse Attention (ProbAttention), with its unique probabilistic sampling mechanism, performs well in later-stage predictions, with relatively small errors in the curves of modes 3, 7, and 9. However, probabilistic sampling may miss some low-frequency but crucial detail information during the process or introduce a certain degree of randomness in some periods, resulting in its overall performance being slightly weaker than that of the standard multi-head attention, as shown in modes 2 and 8. Overall, all three types of Transformers outperform LSTM, but the standard multi-head attention performs slightly better in terms of stability and global dependency modeling.

**Figure 16:**
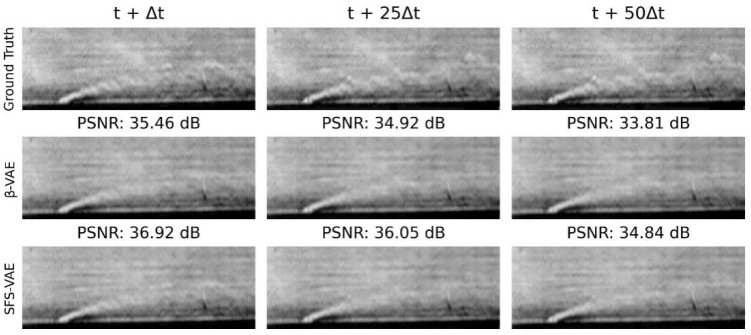
Prediction image results from SFS-VAE and β-VAE on the transformer.

Figure 16 shows the decoding results after using the standard multi-head attention Transformer for latent vector prediction, comparing the flow field image reconstruction performance of the β-VAE and SFS-VAE models at 1 step, 25 steps, and 50 steps. Specifically, at 1-step prediction, the predicted flow field obtained from the latent vector based on β-VAE through the Transformer can reproduce the global features; however, the specific details and jet boundaries are slightly blurred. In contrast, the prediction result based on SFS-VAE exhibits a clearer overall structure and better local details, with a significant increase in PSNR. When the prediction horizon extends to 25 steps, the prediction result of β-VAE begins to deteriorate, with many details lost and the jet tail becoming difficult to observe, while in the SFS-VAE result, although local noise gradually increases, the overall contour and salient features are still relatively well preserved. At 50-step prediction, both models exhibit certain prediction errors. Referring to Figure 15, it can be observed that the errors in SFS-VAE are primarily caused by prediction inaccuracies in latent vectors 1, 2, and 7. Nevertheless, SFS-VAE consistently maintains a higher PSNR than β-VAE. These results indicate that SFS-VAE, by producing more accurate latent representations, effectively improves the stability and fidelity of temporal prediction.

## 5. Parameter Optimization and Generalization Performance

### 5.1. The Impact of Hyperparameters on Performance Indicators

Table 2 reports the performance comparisons between the β-VAE and SFS-VAE models in terms of energy metrics, ${det}_{R}$, average image reconstruction PSNR, and the PSNR at the 50th prediction step under different latent variable dimensions (Dims) and β parameter settings. It can be observed from the table that when the β parameter is small, both models obtain high energy values and image reconstruction metrics. For instance, when the dimension is 10, the β-VAE model reaches 71.3% energy and a reconstruction PSNR of 36.97 at β = 0.001, while the prediction PSNR is 34.40. This indicates that with a lower β penalty, the model is more sensitive to reconstruction errors, thereby achieving better image restoration results. In contrast, as the β increases to 0.01, both the energy and PSNR of the models decrease, but ${det}_{R}$ increases. This is because a larger KL divergence weight suppresses the expressive power of the latent space, causing the model to generate more orthogonally separated reconstructed images when balancing reconstruction and regularization. However, when β is excessively large, ${det}_{R}$ decreases instead. This is because overly strong KL regularization can lead to posterior collapse, where some latent variables become inactive or indistinguishable, and many dimensions may exhibit similar distribution characteristics due to excessive regularization. As a result, the number of effective dimensions decreases, and the information expression overlaps between dimensions intensifies, which in turn reduces the independence between vectors. Additionally, SFS-VAE has the largest ${det}_{R}$ at β = 0.01, while β-VAE shows better independence at β = 0.005. This suggests that for different models, we may need to consider choosing β to balance reconstruction performance and orthogonality.

Regarding the influence of dimensions, we observe that for both β-VAE and SFS-VAE, the energy and ${det}_{R}$ metrics vary as the latent variable dimension increases. For example, in the β-VAE model, the energy and ${det}_{R}$ are 71.3 and 89.23, respectively, when the dimension is 10, but when the dimension is 20, the energy increases while ${det}_{R}$ decreases. This implies that although higher dimensions can capture more energy, they may also introduce redundant information or make it difficult for the model to fully separate key features. In contrast, in the 10-dimensional and 20-dimensional SFS-VAE models, the energy improvement is not significant, especially when β is small. We believe this is because the effective information in the data has been largely captured around a 75% energy level. Due to the characteristics of schlieren data, which has a small data volume and high noise, the upper limit of the effective energy it contains is relatively low, and it cannot capture over 90% of the energy like flow field simulation data. Therefore, in this case, increasing the latent variable dimension to a certain extent and then further increasing it contributes very little to the energy improvement and may even introduce redundant information. This reminds us that in future work, we should consider increasing the memory capacity of the shooting equipment to extend the temporal length of the dataset or generate more data through high-fidelity video frame interpolation models.

Overall, the SFS-VAE model consistently outperforms the β-VAE across most parameter combinations, especially demonstrating superior PSNR in image reconstruction, while not significantly increasing the number of parameters. This balance offers potential for real-time applications.

### 5.2. Analysis of Generalization Capability Under Diverse Operating Conditions

To comprehensively evaluate the generalization capability of the model under diverse operating conditions, we designed additional experiments using a controlled variable approach. The experiment conducted earlier in the text (Mach number 0.1, temperature ratio 1.3, blowing ratio 2.5) is designated as Working Condition 1 and serves as the baseline for comparison. In Working Condition 2, the mainstream Mach number is increased to 0.3 while keeping the temperature ratio (1.3) and blowing ratio (2.5) unchanged, in order to investigate the influence of higher inlet velocities on model performance. In Working Condition 3, the Mach number (0.1) and blowing ratio (2.5) are kept consistent with Working Condition 1, while the temperature ratio is reduced to 1.2 to evaluate the effect of weakened thermal gradients on flow feature reconstruction.

**Figure 17:**
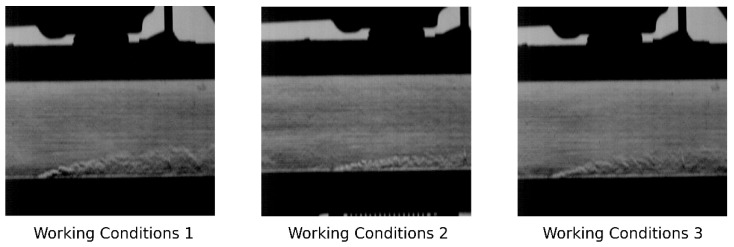
First Frame of Schlieren Images under Working Conditions 1, 2, and 3.

Figure 17 presents the first frame of the schlieren images obtained under each working condition, providing a visual reference for the differences in flow field structures. Table 3 compares the performance of the models under these conditions. In Working Condition 2, increasing the Mach number at the wind tunnel inlet compresses the range of the jet area, making the decoupling task more difficult, and Det_R_ decreases. Although the reconstruction index does not change much, this is attributable to Working Condition 2 having a larger mainstream area, which inflates the metric. In fact, an analysis of the pixel-wise difference map, generated using the same method as in Figure 7, confirms that the reconstruction effect of the jet area has declined. A decrease in the temperature ratio in Working Condition 3 causes the flow structure in the schlieren image to be less defined, which leads to a decline in the reconstruction index. This is because a lower temperature ratio reduces the density gradient in the flow field, thereby weakening the contrast in the schlieren visualization and making it more difficult for the model to extract meaningful flow features.

Overall, for the SFS-VAE model, the changes in various indicators under different working conditions are minimal, with fluctuations within 1 to 2 units, indicating that the model possesses a certain generalization ability. Compared with β-VAE, although ${det}_{R}$ has decreased, the average energy has increased by about 6%, and the PSNR has also increased. This trade-off is acceptable. If a higher ${det}_{R}$ is needed, the β parameter can be appropriately increased. These results demonstrate that the optimized SFS-VAE exhibits robustness in cross-working condition generalization for schlieren flow field data and can better adapt to the challenges posed by environmental parameter variations in the schlieren reconstruction task.

### 5.3. Influence of the Position of the PFSM

**Figure 18:**
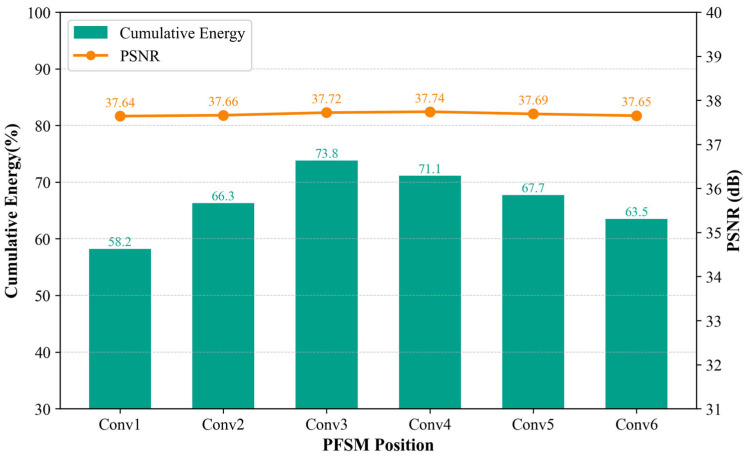
Influence of the PFSM position on the reconstruction performance.

In the experiment exploring the module positions, we discovered an interesting phenomenon. When adjusting the position of the FSEM, the PSNR index and cumulative energy of the reconstruction consistently increased. However, analysis of the PFSM revealed that its placement had a significant impact on overall performance. Specifically, when the PFSM was positioned after the third convolutional block among the six convolutional blocks, both the energy index and PSNR reached their maximum values. Conversely, placing this module after other convolutional blocks, especially near the lower or higher layers of the convolutional network, caused a notable decline in the cumulative energy index, as shown in the results of Figure 18. It is worth noting, however, that despite the significant drop in the energy index, the quality of image reconstruction did not change substantially. We believe this is because the change in module position, although affecting the encoder’s ability to capture flow field information, was compensated for by the decoder, so only the energy index calculated from the latent vector was impacted. This indicates that the selection of the PFSM position plays a crucial role in balancing local frequency response and global feature integration within the network. Placing it in the middle layer enables the network to capture key high-frequency details in the schlieren image at an appropriate intermediate abstraction level, avoiding feature loss or redundancy due to premature or delayed fusion, thereby achieving a better balance between energy concentration and reconstruction quality.

## 6. Conclusions and Discussion

This paper addresses the challenge of efficiently reducing dimensionality and predicting flow field images captured by schlieren photography. It proposes and validates the SFS-VAE model, highlighting its performance and design advantages in the following aspects:

For the reduced-order modeling and reconstruction task, SFS-VAE effectively captures multi-scale features in both frequency and spatial domains by introducing two key modules. The PFSM dynamically enhances flow structures across different frequency bands through discrete Fourier transforms and multi-scale convolution, while the FSEM improves the effective representation of the jet area using a gradient-driven spatial attention mechanism. Experimental results show that compared with the traditional β-VAE, SFS-VAE reduces RMSE by approximately 16.9%, increases PSNR by about 1.6 dB, and achieves a cumulative modal energy of 73.8%. The modal decomposition also demonstrates stronger interpretability, underscoring the advantages of SFS-VAE in feature decoupling and energy extraction.

In the time-series prediction task, this study evaluates multiple Transformer models with different attention mechanisms to model latent vectors, enhancing the stability and accuracy of future flow field evolution predictions. Experimental results reveal that, based on the high-quality reduced-order representations from SFS-VAE, predicted curves closely match real data in short-term time series prediction, validating the model’s robustness and generalization capabilities in long-term prediction tasks. Additionally, the study discusses model parameters and module placement, clarifying key optimization factors. Experiments show that with a latent variable dimension of 10 and with β set to 0.01, the model achieves improved feature orthogonality while maintaining reconstruction accuracy. Regarding PFSM placement, optimal energy accumulation and image reconstruction performance are achieved only when the module is positioned in the middle layer of the encoder. Placement at the bottom or top layers results in a significant decrease in energy despite minimal change in PSNR, indicating a strong correlation between module position and the model’s information extraction ability. Cross-condition experiments verify the strong generalization capability of SFS-VAE across different Mach numbers, temperature ratios, and blowing ratios. The fluctuations in various performance indicators are limited to about 1 to 2 units, demonstrating the model’s broad applicability in engineering flow control and real-time monitoring.

The integration of PFSM and FSEM enhances reconstruction fidelity in sharp-gradient, jet-boundary, and fine-scale regions, improving local detail while preserving the physical interpretability of latent modes. However, several limitations must be acknowledged. First, enhancement performance is sensitive to hyperparameter tuning, including convolution kernel scales, attention weights, and the balance between frequency and spatial contributions. Optimal values can vary with flow regimes and image acquisition settings, necessitating re-tuning or adaptive strategies for deployment in diverse scenarios. Second, while the current model introduces minimal additional training overhead compared with the baseline VAE at moderate resolutions, its inference performance under higher-resolution datasets and longer time-series inputs still requires careful evaluation, which is critical for practical applicability. Due to equipment limitations, experiments involving higher-resolution schlieren datasets, extended temporal sequences, and performance benchmarks across different GPU devices have not yet been conducted, constraining generalization and scalability analysis. Lastly, while the enhancement improves modal interpretability for velocity- and density-related structures, its extension to other scalar fields remains unverified and may require tailored preprocessing or feature fusion schemes. These limitations highlight the need for adaptive parameter control, automated module configuration, more robust denoising, and the development of scalable training and inference strategies.

Ultimately, this work not only provides a powerful tool for fluid dynamics diagnostics but also presents a robust framework for interpreting complex, high-dimensional data across scientific imaging domains. Future research will pursue several directions: (1) developing adaptive β parameter selection strategies that automatically adjust based on latent space energy distribution and reconstruction-error patterns; (2) implementing self-tuning enhancement modules, where PFSM scales and FSEM attention weighting are optimized via meta-learning to minimize manual tuning; (3) introducing enhanced data-augmentation strategies—such as diffusion-model-based frame interpolation—to enrich schlieren image datasets; (4) extending the framework to cases where different physical effects interact, such as flows with chemical reactions or combined air-and-heat effects; (5) conducting systematic performance benchmarking across different GPU architectures to evaluate inference efficiency, optimize resource utilization, and support scalability to higher-resolution datasets and extended temporal sequences. These improvements will not only address the current limitations but also broaden the applicability of SFS-VAE towards more diverse, complex, and operational environments.

## Figures and Tables

**Table 1:** Parameters of the SFS-VAE network.

Encoder Layer	Output Shape	Decoder Layer	Output Shape
Input	(128, 320, 1)	Latent Input	(r)
1st Conv2D	(64, 160, 8)	2nd Dense	(2560)
ELU Activation	(64, 160, 8)	Reshape	(2, 5, 256)
2nd Conv2D	(32, 80, 16)	1st Conv2DTranspose	(4, 19, 128)
ELU Activation	(32, 80, 16)	ELU Activation	(4, 10, 128)
FSEM	(32, 80, 16)	2nd Conv2Dtranspose	(8, 20, 64)
3rd Conv2D	(16, 40, 32)	ELU Activation	(8, 20, 64)
ELU Activation	(16, 40, 32)	FSEM	(8, 20, 64)
PFSM	(16, 40, 32)	3rd Conv2DTranspose	(16, 40, 32)
4th Conv2D	(8, 20, 64)	ELU Activation	(16, 40, 32)
ELU Activation	(8, 20, 64)	PFSM	(16, 40, 32)
FSEM	(8, 20, 64)	4th Conv2DTranspose	(32, 80, 16)
5th Conv2D	(4, 10, 128)	ELU Activation	(32, 80, 16)
ELU Activation	(4, 10, 128)	FSEM	(8, 20, 64)
6th Conv2D	(2, 5, 256)	5th Conv2DTranspose	(64, 160, 8)
ELU Activation	(2, 5, 256)	ELU Activation	(64, 160, 8)
Flatten Layer	(2560)	6th Conv2DTranspose	(128, 320, 1)
1st Dense	(*r*)	Output	(128, 320, 1)

**Table 2:** Influence of hyperparameters on the performance of the β-VAE and SFS-VAE (Values in bold indicate the optimal performance under the corresponding method and dims parameter).

Method	Dims	Para (K)	β	Energy (%)	Det_R_	Reconstruction PSNR (dB)	Prediction PSNR (dB) *
β-VAE	10	2108	0.001	**71.3**	89.23	**36.97**	**34.40**
			0.005	69.8	**92.23**	36.79	34.13
			0.01	67.2	90.64	36.12	33.81
			0.02	65.4	85.06	35.83	33.05
β-VAE	20	2115	0.001	**72.7**	90.54	**37.43**	34.51
			0.005	71.4	**91.81**	37.02	**34.60**
			0.01	70.5	88.67	36.85	34.26
			0.02	65.9	83.40	35.92	32.99
SFS-VAE	10	2136	0.001	**74.0**	85.93	**37.76**	34.91
			0.005	73.8	87.02	37.74	**34.97**
			0.01	73.8	**88.71**	37.72	34.84
			0.02	69.9	83.36	36.83	34.26
SFS-VAE	20	2144	0.001	**74.5**	85.34	**37.95**	**35.14**
			0.005	74.1	86.06	37.83	34.82
			0.01	74.1	**87.18**	37.82	34.88
			0.02	70.6	81.59	36.81	34.29

* Prediction PSNR is the PSNR at the 50th prediction step.

**Table 3:** Results of the β-VAE and SFS-VAE under three working conditions (Values in bold indicate the optimal performance under the corresponding method).

Method	Conditions	Energy (%)	Det_R_	Reconstruction PSNR (dB)	Prediction PSNR (dB) *
β-VAE	1	**67.2**	90.64	**36.12**	33.81
	2	66.5	**91.23**	35.95	33.62
	3	66.9	90.49	36.07	**33.90**
SFS-VAE	1	**73.8**	**88.76**	**37.72**	**34.84**
	2	73.3	88.05	37.55	34.47
	3	73.4	87.88	37.58	34.61

* Prediction PSNR is the PSNR at the 50th prediction step.

## Data Availability

The data presented in this study are available on request from the corresponding author, Ronghua Yang.

## Abbreviations

The following abbreviations are used in this manuscript:

ROM	Reduced-Order Model
POD	Proper Orthogonal Decomposition
DMD	Dynamic Mode Decomposition
CAE	Convolutional Autoencoder
LSTM	Long Short-Term Memory
RNN	Recurrent Neural Network
FAE	Fully Connected Autoencoders
VAE	Variational Autoencoder
β-VAE	β-Variational Autoencoder
SFS-VAE	Spatial-Frequency-Scale VAE
PFSM	Progressive Frequency-enhanced Spatial Multi-scale Module
FSEM	Feature-Spatial Enhancement Module
FFT	Fast Fourier Transform
IFFT	Inverse Fast Fourier Transform
DWConv	Depthwise Separable Convolution
